# Supplementary material for: Identification of photoperiod-induced specific miRNAs in the adrenal glands of Sunite sheep (Ovis aries)
Source: Front Vet Sci. 2022 Jul 22;9:888207. doi: 10.3389/fvets.2022.888207 (PMC9354845; doi:10.3389/fvets.2022.888207)
Supplement: Supplementary file 1 [file Data_Sheet_1.ZIP › Supplementary file/miRDeep2 file.zip!/miRDeep2 file/Know_Structure/LP42A1/oar-mir-362.pdf]

miRBase precursor	: oar-mir-362
Total read count	: 1582
oar-miR-362 read count	: 1546
remaining reads	: 36

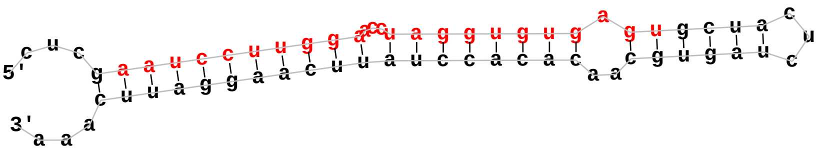

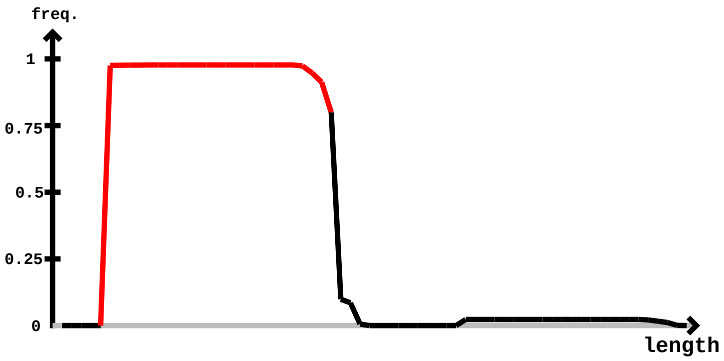


```
      oar-miR-362
5'- cucgaauccuuggaaccuaggugugagugcuacucuagugcaacacaccuauucaaggauucaaa   -3'    exp
    ...((((((((((...((((((((.((((((...))))))..))))))))))))))))))...    reads      mm         sample
    ....aauccuuggaaccuaggugu.........................................    6          0          seq
    ....aauccuuggaaccuaggugug........................................    41         0          seq
    ....aauccuuggaaccuagguguga.......................................    55         0          seq
    ....aauccuuggaaccuaggugugag......................................    179        0          seq
    ....aauccuuggaaccuaggugugagu.....................................    1107       0          seq
    ....aauccuuggaaccuaggugugagug....................................    21         0          seq
    ....aauccuuggaaccuaggugugagugc...................................    125        0          seq
    ....aauccuuggaaccuaggugugagugcu..................................    9          0          seq
    .....auccuuggaaccuaggugugagu.....................................    1          0          seq
    ......uccuuggaaccuaggugugagugc...................................    1          0          seq
    ........cuuggaaccuaggugugagu.....................................    1          0          seq
    .........................................aacacaccuauucaaggau.....    3          0          seq
    .........................................aacacaccuauucaaggauu....    7          0          seq
    .........................................aacacaccuauucaaggauuc...    8          0          seq
    .........................................aacacaccuauucaaggauuca..    17         0          seq
    .........................................aacacaccuauucaaggauucaa.    1          0          seq
```
